# Bone-anchored hearing system, contralateral routing of signals hearing aid or cochlear implant: what is best in single-sided deafness?

**DOI:** 10.1007/s00405-021-06634-7

**Published:** 2021-02-10

**Authors:** Till F. Jakob, Iva Speck, Ann-Kathrin Rauch, Frederike Hassepass, Manuel C. Ketterer, Rainer Beck, Antje Aschendorff, Thomas Wesarg, Susan Arndt

**Affiliations:** Department of Oto-Rhino-Laryngology, Medical Center – University of Freiburg, Faculty of Medicine, University of Freiburg, Germany, Killianstraße 5, 79106 Freiburg, Germany

**Keywords:** Single-sided deafness, Cochlear implant, Bone anchored hearing system, Contralateral routing of signals hearing aids hearing aid, SSQ

## Abstract

**Purpose:**

The aim of the study was to compare long-term results after 1 year in patients with single-sided deafness (SSD) who were fitted with different hearing aids. The participants tested contralateral routing of signals (CROS) hearing aids and bone-anchored hearing systems (BAHS). They were also informed about the possibility of a cochlear implant (CI) and chose one of the three devices. We also investigated which factors influenced the choice of device.

**Methods:**

Prospective study with 89 SSD participants who were divided into three groups by choosing BAHS, CROS, or CI. All participants received test batteries with both objective hearing tests (speech perception in noise and sound localisation) and subjective questionnaires.

**Results:**

16 participants opted for BAHS-, 13 for CROS- and 30 for CI-treatment. The greater the subjective impairment caused by SSD, the more likely patients were to opt for surgical treatment (BAHS or CI). The best results in terms of speech perception in noise (especially when sound reaches the deaf ear and noise the hearing ear), sound localization, and subjective results were achieved with CI.

**Conclusion:**

The best results regarding the therapy of SSD are achieved with a CI, followed by BAHS. This was evident both in objective tests and in the subjective questionnaires. Nevertheless, an individual decision is required in each case as to which SSD therapy option is best for the patient. Above all, the patient's subjective impairment and expectations should be included in the decision-making process.

## Introduction

People who suffer from single-sided deafness (SSD) have difficulty understanding speech, especially when the sound comes to the deaf ear or in noise, as well as in the localization of sounds [1]. Patients with SSD should be advised in detail about the various options for hearing rehabilitation. In particular, the limitations as well as the advantages and disadvantages of the different devices should be discussed. In addition to the avoidance of technical support with the aim of developing compensatory mechanisms (especially for sound localisation when hearing loss occurred in childhood; [2]), there are three different options for rehabilitation of the deaf ear based on a device. Over the last few years, the treatment with best results for unilateral deaf patients has been cochlear implant (CI; [3–5]), whenever possible and desired by the patient. Alternative treatment options for SSD are contralateral routing of signals hearing aids (CROS) [6] or bone-anchored hearing systems (BAHS) [7]. The BAHS can be worn on a headband, transcutaneously or percutaneously. Binaural hearing is possible with neither CROS nor BAHS and no beneficial effect regarding speech perception in noise and sound localisation is shown in a review [8] although one study showed an improvement in speech-in-noise performance for SSD [9]. Advantages of a CI are the restoration of binaural hearing and the associated better speech perception in noise and better sound localisation [4]. Aim of this study was to investigate benefits of different hearing rehabilitation options in SSD focusing on: Which device (BAHS, CROS, or CI) provides the best results in objective hearing tests after 12 months of using the selected device? Based on questionnaires, what is the subjective benefit of the individual devices? What are the factors that may influence the decision for a particular hearing device? Are there differences between immediate testing and in long-term use after 12 months?

## Materials and methods

### Study design and participants

In this prospective study, 89 adult (age > 18 years) patients with SSD were included. The study was conducted in accordance with the recommendations of the Ethics Committee of the University of Freiburg, Germany (protocol numbers 175/08 and 69/09) and in accordance with the Declaration of Helsinki (2013). The audiological classification of SSD was: untreated hearing ability of the poorer-hearing ear with a mean bone-conduction audiometric threshold for the frequencies 500, 1000, 2000, 4000 Hz (bone-conduction four-frequency pure-tone average, BC 4PTA) greater than or equal to 70 dB hearing level (HL) and an air-conduction 4PTA of less than or equal to 30 dB HL for the better-hearing ear [10]. The first part of the study is a prospective descriptive cohort study with a randomised 3-week test period of BAHS and CROS devices. The second part of the study is the 12-month follow-up after choosing the treatment option (BAHS, CROS or CI).

After signed informed consent was obtained, an audiological assessment was performed in all participants at baseline including the following measures: pure-tone hearing thresholds, speech intelligibility for Freiburg monosyllables and numbers, tympanograms, and auditory brainstem responses (ABR). In participants who decided for a CI, transtympanal electrocochleography or promontorial testing, temporal bone CT and MRI were performed. After the test periods of randomised 2 × 3 weeks (3-week BAHS-testing on head band and 3-week CROS-testing) the participants were divided into three groups: Group A: participants who decided on a BAHS, group B: participants who decided on a CROS hearing aid, and group C: participants who decided on a CI. The participants of groups A and C were then implanted (usually BAHS under local anaesthesia and CI under general anaesthesia). The outcomes of some patients were discussed in previous publications [4, 5].

### Study Plan

**Fig. 1:**
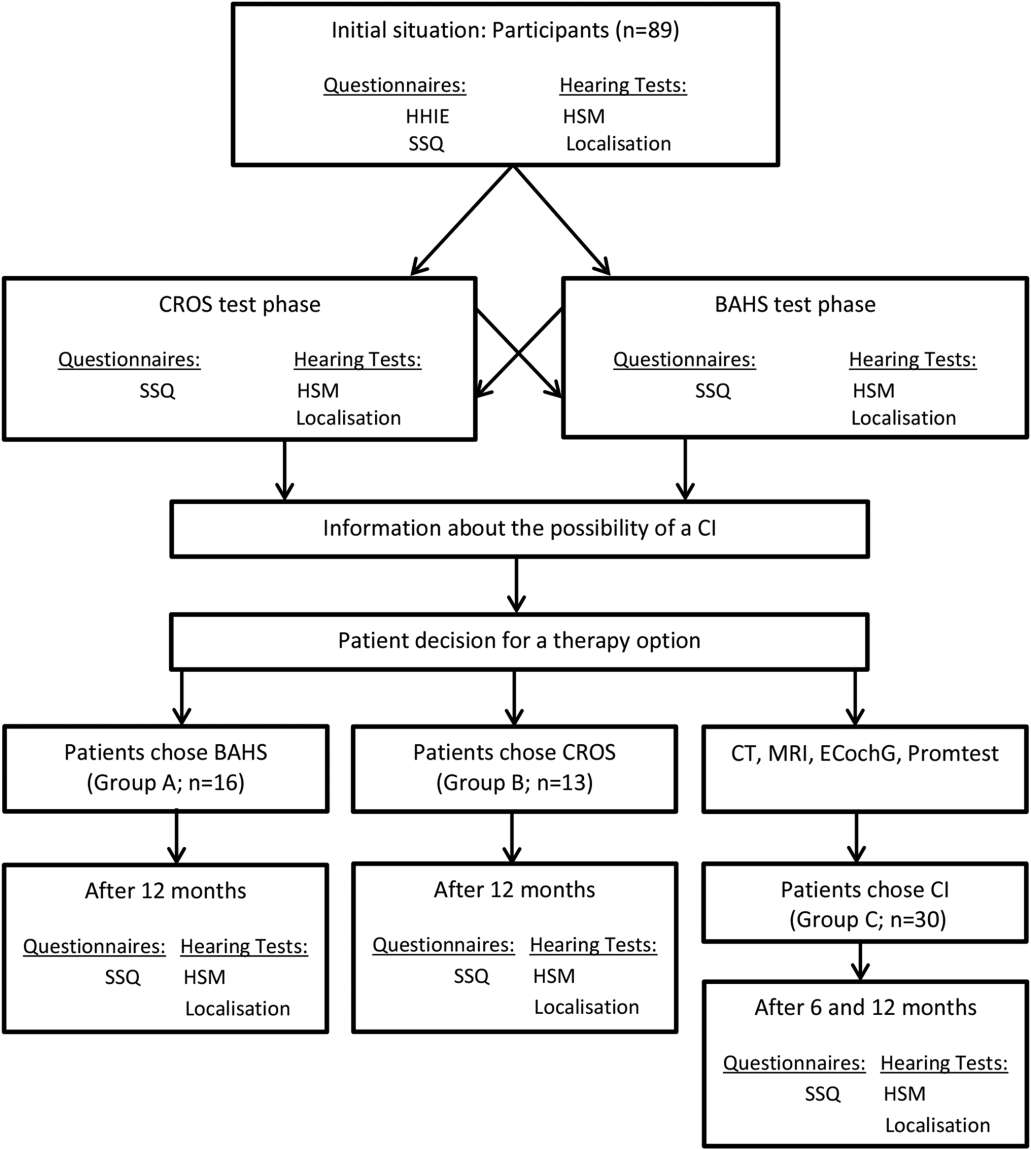
Flowchart of study design: Of the initial 89 participants, 59 participants decided to continue the study. 16 participants underwent BAHS surgery, 13 chose CROS hearing aids and 30 participants underwent CI surgery. The times of the hearing tests and questionnaires are shown in the flowchart. *BAHS* bone-anchored hearing system, *CI* cochlear implant, *CROS* contralateral routing of signals, *ECochG* Electrocochleography, *HHIE* Hearing Handicap Inventory for Adults, *HSM* Hochmair–Schulz–Moser sentence test, *IOI-HA* International Outcome Inventory for Hearing Aids, *OLSA* Oldenburg sentence test, *Promtest* promontorial testing, *SSQ* Speech, Spatial and Qualities of Hearing Scale

All study participants completed a 3-week test phase with CROS and BAHS. Which device was tested first was determined randomly. After each of the test phases, the participants filled out questionnaires in which their subjective experiences were recorded. Hearing tests were also performed to determine the objective hearing ability. In addition, all participants who were in principle eligible for a CI were informed about the possibility of CI treatment. After the test phase, the participants could decide whether they wanted to continue participating in the study and what kind of hearing rehabilitation they wanted. Therefore, the number of participants in each group is different. The tests were repeated after 12 months for the participants in groups A and B. For group C participants, the tests were repeated after 6 and after 12 months. Testing after 6 months was performed routinely as part of rehabilitation (Fig. 1).

### Test battery

Speech in noise testing: Speech intelligibility in noise was assessed using the Hochmair–Schulz–Moser (HSM) sentence test [11]. Speech and background noise were presented at 65 dB SPL, i.e., with a fixed signal-to-noise ratio (SNR) of 0 dB. Three speech (S) in noise (N) presentation conditions were examined: S0N0, SnhNssd and SssdNnh (nh, normal hearing side; ssd, single-sided deafness side). In presentation condition S0N0, both speech and noise were presented from the front at an angle of 0°. In the presentation condition SnhNssd, speech was presented from the normal-hearing side and background noise from the deaf side at an angle of + 45° and -45°, and vice versa in the presentation condition SssdNnh (speech from the deaf side and background noise from the normal-hearing side each at an angle of 45°; setup according to Arndt et al. [4, 12]). The tests were performed at the beginning of the study without any hearing aid, after a 3-week test trial with BAHS and after a 3-week test trial with CROS, and after 12 months with the supplied device (in case of CI also after 6 months; see Fig. 1).

Localisation: Localisation abilities were tested in a sound-isolated room with seven loudspeakers positioned in a frontal semicircle 2 m in diameter with a distance of 30° in the horizontal plane around the participant at head level. Sentences of the Oldenburg sentence test (OLSA) [13] were used as stimuli for the assessment of localisation ability. In each localisation test, 70 sentences (10 per loudspeaker) were presented at sound levels of 59, 62, 65, 68 and 71 dB SPL and a mean sound level of 65 dB SPL, in random sequence from one of the 7 loudspeakers. For each participant and each condition, the localisation ability was measured as the angle error in degrees, that is, as the mean difference in angle between the presentation loudspeaker and the loudspeaker identified by the patient (setup according to Arndt et al. [4, 12]).

### Questionnaires

Hearing Handicap Inventory for Adults (HHIA): This ten-item questionnaire records the subjectively perceived limitation on a social and emotional level that the patient brings into the study due to his single-sided deafness [14]. Since the questionnaire was intended to capture the initial situation, it was only applied at the beginning of the study. The degree of subjective impairment is divided into three groups: no hearing handicap (0–8 points), mild-to-moderate hearing handicap (10–24 points) and significant hearing handicap (26–40 points).

Speech, Spatial and Qualities of Hearing Scale (SSQ): This 3-section questionnaire assesses speech understanding, spatial hearing, and hearing quality with a scoring system of 0 to 10 for each item. Unable to hear is represented by 0 and 10 means hears perfectly [15].

### Data Analysis

Statistical analysis was carried out in GNU R (R Core Team, 2014) and illustrated by Box–Whisker plots. The Shapiro–Wilk test was used to check the data for normal distribution. The Kruskal–Wallis test was performed for comparison of the three groups (A–C) in SSQ, localisation ability and speech recognition in noise. For post hoc analyses, pairwise comparison with the Wilcoxon-rank tests using Bonferroni correction was applied. A level of significance of 0.05 was applied in all analyses.

## Results

### Participants

In this study, 89 adult participants were included with an average age of 55.8 years and an average period of single-sided deafness of 10.7 years (1 month to 51.1 years; average age and period of SSD for each group, see Table 1). There was no significant difference in age between the groups (*p* = 0.359). However, we found a difference between the groups in the duration of deafness (*p* = 0.007). The duration of deafness was shorter for group C than in the other groups (Group A vs. Group C: *p* = 0.004; Group B vs. Group C: *p* = 0.029; no treatment vs. Group C: *p* = 0.008). After the two trials, 16 participants chose BAHS (Group A; implants: 12 Cochlear™ Baha BP100®, 3 Oticon Medical™ Ponto Power®, 1 Cochlear™ Baha Intenso®), 13 participants chose CROS (Group B; Phonak Una M), and 30 participants chose CI (Group C; implants: 17 Cochlear™ Nucleus® CI512, 12 Cochlear™ Nucleus® CI24RE, 1 Cochlear™ Nucleus® CI422). 20 participants decided against treatment and 10 participants stopped the study. The main reason for SSD was sudden hearing loss (*n* = 29), followed by unclear reason of SSD since childhood (*n* = 13). For further aetiologies, see Table 1.

**Table 1:** Demographic characteristics per group

Group	No treatment	BAHS (group A)	CROS (group B)	CI (group C)
Number	20	16	13	30
Gender	f: *n* = 13 m: *n* = 7	f: *n* = 6 m: *n* = 10	f: *n* = 5 m: *n* = 8	f: *n* = 15 m: *n* = 15
Age (mean ± SD)	55.0 ± 13.1 years	60.8 ± 13.9 years	56.8 ± 15.4 years	53.4 ± 12.3 years
Deafness duration (mean ± SD)	187.6 ± 197.3 months	158.3 ± 186.1 months	221.8 ± 227.6 months	34.7 ± 45.9 months
4PTA better ear (mean ± SD)	13.5 ± 8.4 dB	17.8 ± 12.6 dB	16.6 ± 10.8 dB	15.6 ± 14.4 dB
PTA poorer ear (mean ± SD)	100.8 ± 20.8 dB	99.6 ± 21.6 dB	116.1 ± 21.7 dB	104.7 ± 22.3 dB
Aetiology: Sudden idiopathic hearing loss	8	4	4	13
Unknown since childhood	5	3	5	0
Vestibular schwannoma	1	4	3	0
Labyrinthitis	0	0	1	5
Menière´s disease	2	0	0	1
After ear surgery	1	2	0	3
Otosclerosis	0	1	0	3
Meningitis	2	0	0	1
Temporal bone fracture	1	0	0	1
Mumps	0	1	0	1
Otitis media	0	1	0	1
Cogan-1-Syndrome	0	0	0	1

### Results of HHIA

Of the participants, 5.5% (3 of 55) felt no hearing handicap (0–8 points), 40.0% (22 of 55) felt a mild-to-moderate hearing handicap (10–24 points) and 54.5% (30 of 55) a significant hearing handicap (26–40 points). Four patients did not return the questionnaire. Group A rated their hearing handicap on average with 26.8 points (SD 9.6), Group B with 18 points (SD 8.9) and Group C with 28.2 points (SD 9.9). BAHS (9 of 15) and CI (19 of 29) were mostly chosen by participants with severe impairment and CROS (8 of 11) by participants with mild-to-moderate impairment.

### Test batteries

#### Speech in noise (HSM sentence test)

**Fig. 2:**
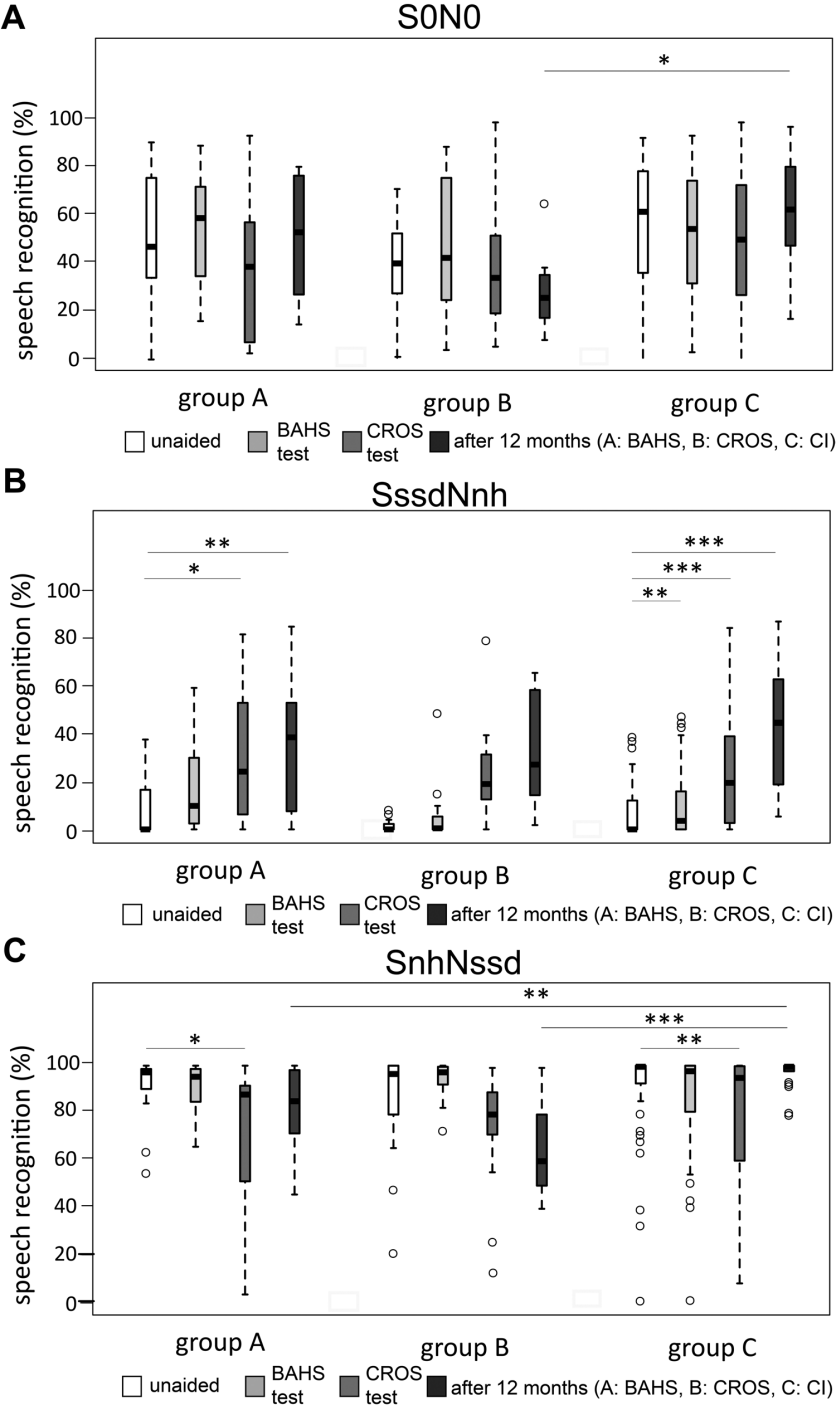
Box–Whisker plots of Hochmair–Schulz–Moser (HSM) sentence test: **a** S0N0 (S: speech; N: noise) presentation setup with speech and noise from the front. Group A: participants who chose BAHS; group B: participants who chose CROS; group C: participants who chose CI. **b** SssdNnh (ssd: single-sided deafness; nh: normal hearing) presentation setup with speech from the unilateral deaf side, noise from the normal hearing side. **c** SnhNssd presentation setup with speech from the normal hearing side, noise from the unilateral deaf side. *BAHS* bone-anchored hearing system, *CI* cochlear implant, *CROS* contralateral routing of signals

Results of HSM sentence test at different time points, different treatment options, and different presentation configurations are shown in Fig. 2A–C.

Group A: After the 3-week test period with a headband, no significant improvements for BAHS were found in all test configurations (S0N0: *p* = 0.4351, SssdNnh: *p* = 0.06921, SnhNssd: *p* = 1; Fig. 2a–c). Although not significant, there is a trend in the SssdNnh situation (median untreated = 0%; median BAHS = 5.66%; Fig. 2b). After CROS testing, the SssdNnh situation showed a significant improvement compared to the untreated situation (*p* = 0.003; median unaided = 0%; median CROS = 27.83%; Fig. 2b). In the SnhNssd situation, however, a significant deterioration in speech understanding was observed when using a CROS (p = 0.010; median unaided = 97%; median CROS = 81%; Fig. 2c). After implantation of the BAHS and testing after 12 months, there was a significant improvement in the SssdNnh configuration (*p* = 0.008; median unaided = 0%, median BAHS 12 m = 40.59%; Fig. 2b).

Group B: After the 3-week test period with the CROS system, no significant improvements were found in all test configurations (S0N0: *p* = 0.570; SssdNnh: *p* = 0.151; SnhNssd: *p* = 0.101; Fig. 2a–c). No differences were found after BAHS testing, either (S0N0: *p *= 0.570; SssdNnh: *p* = 1; SnhNssd: *p* = 1; Fig. 2a–c). After 12 months there was also no improvement with CROS. Although not significant, CROS seems to be associated with poorer speech comprehension compared to the unaided situation in the SnhNssd configuration (median unaided = 98.58%; median CROS = 79.25%, *p* = 0.101; median CROS 12 m = 64.62%, *p* = 0.603).

Group C: BAHS testing, CROS testing and CI after 12 months showed no differences in the S0N0 (*p* = 0.088) configuration compared to the untreated situation. In the SssdNnh configuration, all 3 devices significantly improved speech understanding (unaided vs. BAHA: *p* = 0.005; unaided vs. CROS: *p* < 0.001; unaided vs. CI 12 months: *p* < 0.001). In the SnhNssd configuration, the CROS resulted in a significant decrease in speech understanding compared to the unaided situation (unaided vs. CROS: *p* = 0.001).

Comparing the 12-month results between the three groups showed significantly better speech perception with CI in the S0N0 configuration compared to the CROS group (*p* = 0.017) and better speech perception in the SnhNssd configuration compared to BAHS group (*p* = 0.002) and CROS group (*p* < 0.001).

#### Localisation

**Fig. 3:**
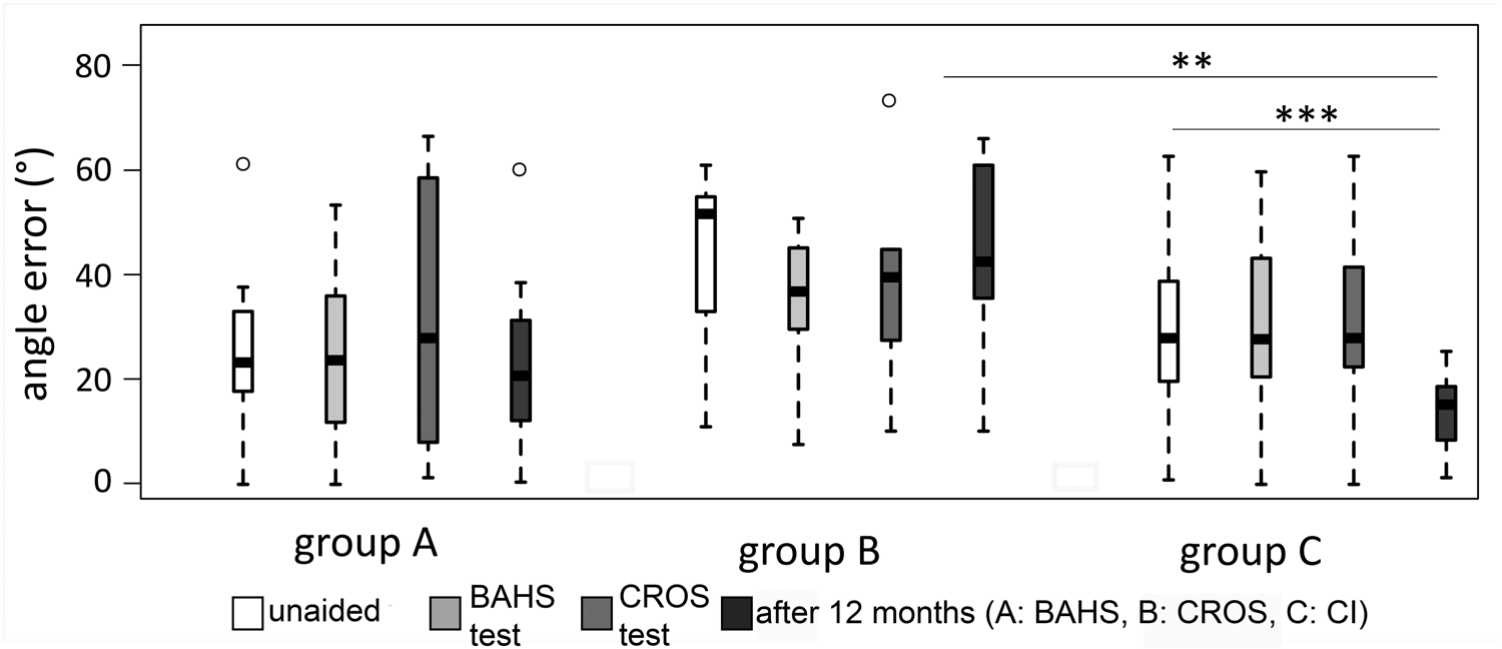
Box–Whisker plots of localisation error: group A: participants who chose BAHS; group B: participants who chose CROS; group C: participants who chose CI. *BAHS* bone-anchored hearing system, *CI* cochlear implant, *CROS* contralateral routing of signals

Localisation errors of all three groups are shown in Fig. 3. Localisation error is significantly reduced in the CI group after 12 months by 10.93° (median unaided 26.36°, median CI 12 m = 15.43°; *p* < 0.001) compared to the unaided conditions. No differences were found in Groups A and B. Comparing the 12-month results there was a significant difference between Group B and C (*p* = 0.008; A vs. B: *p* = 0.095; A vs. C: *p* = 0.151).

### SSQ 

**Fig. 4:**
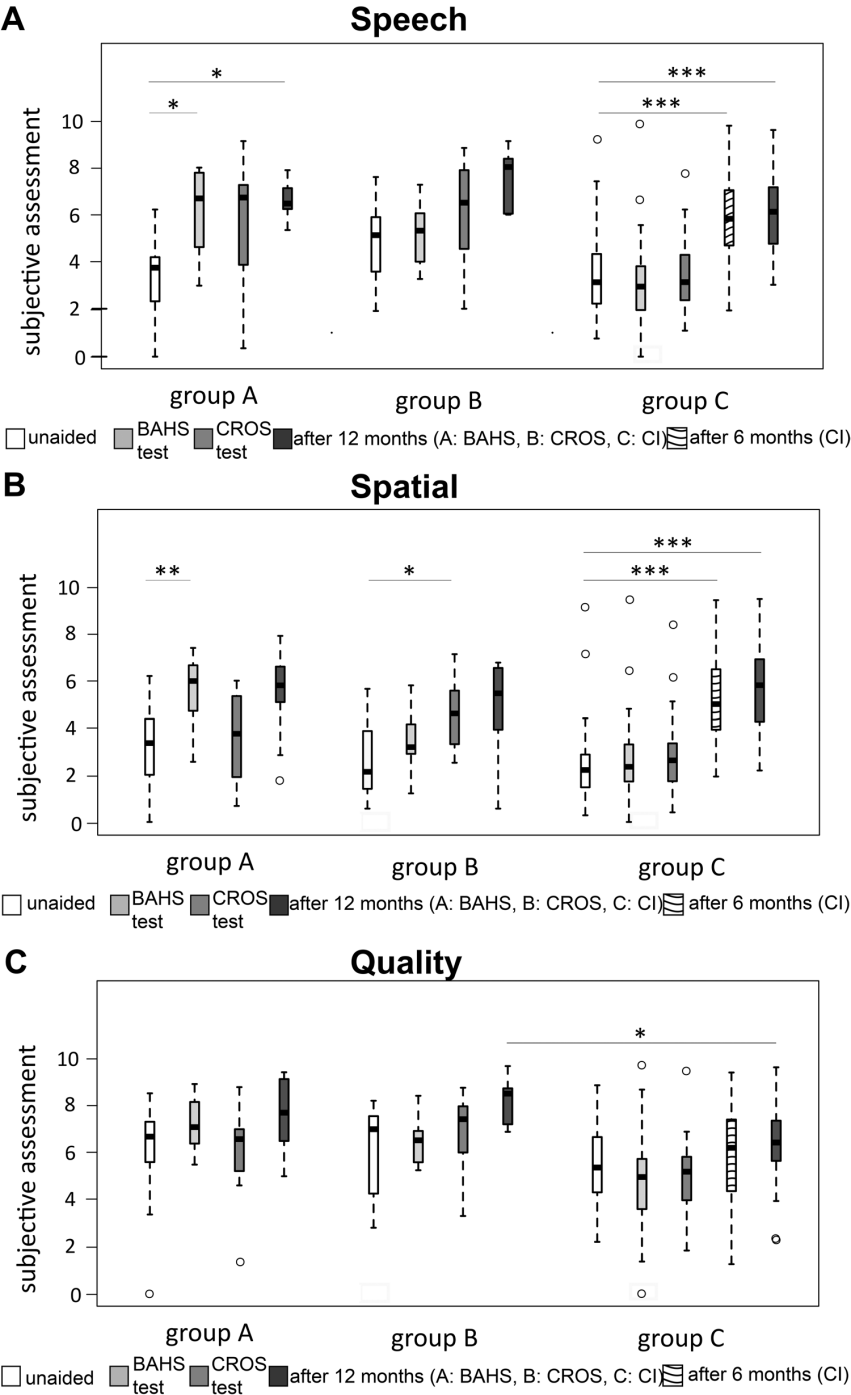
Box–Whisker plots of Speech, Spatial and Qualities of Hearing Scale (SSQ): group A: participants who chose BAHS; group B: participants who chose CROS; group C: participants who chose CI. a Speech understanding; b Spatial hearing; c Hearing quality: For a–c Unable to hear is represented by 0 and 10 means hears perfectly. *BAHS* bone-anchored hearing system, *CI* cochlear implant, *CROS * contralateral routing of signals

Speech understanding: In the speech section there is a significant improvement between the unaided situation (median 3.75) and group A test phase (median 6.61; *p* = 0.024) and after 12 months (median 6.39; *p* = 0.037). No difference was found between the unaided situation (median 5.07) and group B after the CROS test phase (median 6.42; *p* = 0.505) and after 12 months (median 6.94; *p* = 0.236). A significant difference was found between the unaided situation (median 3.05) and group C after 6 months (median 5.54; *p* < 0.0001) and after 12 months (median 5.92; p < 0.0001). In all groups no differences were found between the test phase and after 12 months. No differences were found between the three groups after 12 months (*p* = 0.392; Fig. 4A).

Spatial hearing:

In the spatial section of group A, differences were found between the unaided situation (median 3.5) and after the test phase (median 6.48; *p* = 0.008) and, although not significant, between the unaided situation and after 12 months (median 6.0; *p* = 0.057). Also in group B, differences were found between the unaided situation (median 2.21) and after the test phase (median 4.76; *p* = 0.015) but not between the unaided situation and after 12 months (median 5.65; *p* = 0.236). In group C, differences were found compared to the unaided situation (median 2.26) after 6 (median 5.18; *p* < 0.001) and after 12 months (median 5.83; *p* < 0.001). In all groups no differences were found between the early test phase and after 12 months. No differences were found between the three groups (Fig. 4B).

Hearing quality: In the quality section a difference was only found between group B (8.51) and group C (3.36) after 12 months (*p* = 0.036; Fig 4C).

## Discussion

Based on hearing tests and questionnaires, we compare in this study the results of three different treatment options for SSD, namely BAHS, CROS, and CI. Our results show an improvement in speech comprehension in the situation, where speech comes to the deaf ear and noise to the hearing ear for BAHS and CI. However, there is a negative effect using a CROS in the situation, where speech is applied to the hearing ear and noise to the deaf ear. The localisation of sounds can only be improved by a CI. Subjectively, there is a significant improvement after 12 months in speech understanding and spatial hearing in CI and in speech understanding in BAHS.

In terms of age, the groups were homogeneous, whereas there were differences in the duration of deafness. The shorter duration of deafness in the CI group can be explained by the fact that a CI is not a treatment option in cases of unknown deafness since childhood. In addition, in the case of labyrinthitis, which requires rapid CI implantation due to the risk of cochlear ossification, most patients chose a CI (5 vs. 1 in all other groups combined). In contrast to our 2011 study [4], we included not only patients in whom CROS and BAHS were not successful in the study. Since the patients in the recent study had the choice of which device to use, the group sizes are different. It is noticeable that patients with severe subjective limitations (high score in HHIA questionnaire) tended to opt for surgical therapy like BAHS and CI, whereas patients with less severe limitations tended to opt for a CROS hearing aid. This can be explained by the fact that patients with less subjective impairment from SSD do not want to take the effort and risk of surgery. In addition to the advantage that no surgery is required for a CROS hearing aid, it can also be easily removed and there is no implant or screw in the body. However, of the 13 patients who opted for CROS, only 6 patients came back for follow-up after 12 months. In the CI group all 30 and in the BAHS group all 16 could be re-tested after 12 months. This leads to the assumption that patients with a subjectively low hearing handicap who choose CROS are more likely to be non-users.

Numerous studies have shown improved speech perception and sound localization with a CI in SSD compared to untreated situations [3, 16] or to CROS and BAHS [4, 17]. However, these studies did not look at the results after 1 year. Also, the BAHS were worn with a headband and had not been implanted. The hearing results achieved with a headband are worse than with semi-implantable devices [18]. In the BAHS group, the SssdNnh configuration showed an improvement after 1 year with the implanted BAHS that seems to be better than after 3-week testing with a headband. In this configuration, speech comprehension of HSM sentences improved from 0% (unaided) and 6% after test trial with a headband to 40% with a percutaneous implanted device after 12 months.Similar results are found in a study, where speech perception with an anchored BAHS is found to be 14–20% better than with a headband [19]. Compared to other studies [20], no significant negative effect of the BAHS is found in the NssdSnh configuration. The CI does not negatively influence the situation, either. However, this negative effect is seen with a CROS in a reduction of speech comprehension from 99% unaided to 79% after the test phase and further to 65% (both not significant) after 1 year. In the situation, where noise affects the better ear, a meta-analysis was able to show that a negative effect occurs especially in CROS, followed by BAHS [21]. Comparing the HSM results of the three devices after 1 year, the CI group seems to have the best speech comprehension in all three tested configurations (HSM 12 m S0N0: BAHS: 53%; CROS 29%, CI: 63%; SssdNnh: BAHS = 41%, CROS = 18%, CI = 47%; SnhNssd: BAHS = 84%, CROS = 65%, CI = 99).

Sound localisation was best with CI, with a reduction of the localisation error of 10.93° compared to the unaided situation. The improvement to localise sound by a CI has been shown in numerous studies (for reviews see: [21–23]). We found no benefits regarding sound localisation for the BAHS or CROS group, this is also shown in other studies regarding sound localization (for reviews see: [8, 24]). Other studies even showed significant deficits in localisation performance for BAHS [25] and for CROS [26].

Based on the SSQ questionnaire, speech understanding is improved by BAHS and CI, but not by CROS. Spatial hearing improves subjectively after 12 months only with CI. Hearing quality did not improve statistically with the three devices. Hearing-related quality of life can be measured by the SSQ scale and by the abbreviated profile of hearing aid benefit (APHAB). The latter measures ease of communication, background noise, reverberation and aversion to loud sounds. A meta-analysis found significant benefits for BAHS for all subscales of the APHAB except aversion to loud sounds, for CROS significant benefits were only found for background noise and reverberation. A meta-analysis for SSQ did not identify significant effects of BAHS or CROS compared to the unaided situation, but significant decreases in listening difficulty with CI was found on all subscales [21]. In our previous study, we showed an advantage of the CI in the SSQ over BAHS and CROS, but that was one group that tested all 3 devices and only participants were enrolled whose conventional therapy with CROS and BAHS had not been successful [4].

## Limitations

A disadvantage of the study is the greater ceiling effect when using the HSM with fixed noise and speech level instead of an adaptive procedure. However, since the same test was performed in all three groups, the groups are comparable. A further disadvantage is the different size of the groups, which cannot be avoided, because the patient is allowed to choose the device and is not randomly assigned to a group. Therefore, the groups are not randomized, which results in a further inevitable bias.

## Conclusion

In summary, the best results regarding therapy of SSD are possible with a CI, followed by BAHS. This was evident both in objective tests and in the subjective questionnaires. Nevertheless, an individual decision is required in each case as to which therapy option for SSD is best for the patient. Above all, the patient's subjective impairment and expectations should be included in the decision-making process.
